# Will advancement in technologies bring fear and damage human employment? Evidence from China’s manufacturing industry

**DOI:** 10.1371/journal.pone.0295942

**Published:** 2024-04-26

**Authors:** Peng Wang, Donghai Li, Yangzi Wang, Qingjiang Han, Yousaf Ali Khan

**Affiliations:** 1 School of Business Administration, Henan Polytechnic University, Jiaozuo, Henan, China; 2 School of Government Management, Inner Mongolia Normal University, Hohhot, Inner Mongolia, China; 3 School of Business, Shandong University, Weihai, China; 4 School of Applied Economics, Jiangxi University of Finance and Economics, Nanchang, China; 5 Department of Mathematics and Statistics, Hazara University Mansehra, Mansehra, Pakistan; Zhanjiang University of Science and Technology, CHINA

## Abstract

Advancement in technologies such as robotic industries and artificial intelligence bring fear among human being that jobs will be substituted by robots. Base on the panel data of 28 China’s manufacturing industries, this research analyzed the impact of technical progress bias on employment. First, we calculate the technical progress bias index of 28 industries base on the stochastic frontier model with transcendental logarithm function found 16 industries were toward the skilled labor while the remaining 12 industries were toward the unskilled labor. Second, the empirical results show that technical progress bias has a positive impact on the total manufacturing employment and significant positive effect on the unskilled labor, while no significant impact on skilled labor employment. Third, the threshold effect test proves that if taking industry value-added per capita or R&D capital stock as threshold variable, the threshold about the impact exist, making the impact on skilled labor was insignificant.

## 1. Introduction

Rapid progress of technical progress such as industrial robot, artificial intelligence causes the fear that jobs will be replaced by robots. Will technical progress damage employment? The answers are different. The biased technical progress is considered an important factor affecting employment. Hicks [1] pointed out technical progress is not always neutral, but tend to a certain production factor with lower price. Hicks’s idea inspired later research on the mechanism of the biased technical progress. Many researches focus on the measurement of technical progress bias and studying its relationship with labor market. Kennedy [2] believed that technical progress could save both labor and capital. There is a certain relationship between labor savings and capital savings which Kennedy calls "innovation possibility frontier." Acemoglu [3] gave a strict mathematical definition to technical progress bias. He integrated the endogenous growth theory into the theoretical model, giving it a solid micro foundation. Afterwards, scholars have conducted a large number of empirical tests on the biased technical progress and used them to explain the labor market change. Klump et al. [4] measured the bias of technical progress between capital and labor in the United States and found its capital biased. Autor et al. [5] found the biased technical progress provides assistance for high-skilled jobs and substitutes for middle-skilled jobs, but has little impact on low-skilled jobs, causing labor market polarization. Acemolgu [6] established a task-based model involving the theory of comparative advantage and explains the flow of medium skilled labor. In the second half of the 20th century, employment differentiation and skill premium appeared in the labor market of European and American [7, 8]. It is generally believed that technical progress bias is towards skilled labor, so the skilled labor is more adapted to new technology (especially the computer information technology). Under the technical progress, the demand of skilled labors is more than unskilled labors, making the salary and employment of skilled labors increase. Some studies have formalized this explanation gradually and developed a canonical model [9–11]. The mainstream view represented by the canonical model consider the skill premium is caused by the technical progress bias. At the same time, the labor market of developed countries in Europe and the United States was polarized. Compared with medium-skilled labor, the employment (income) of high-skilled labor and low-skilled labor was rising [12–14]. For the reasons, Autor and Dorn [15] pointed out that the polarization of the labor market was caused by a special type of technical progress, namely, computer and communication technical progress, which was characterized by providing assistance for high-skilled jobs and replacing middle-skilled jobs (streamline work), but had little impact on low-skilled jobs. Therefore, such technical progress tends to favor high-skilled labor over middle-skilled labor. Scholars from China also devote many researches on this issue, but mainly focus on the relationship between technical progress bias and skill premium [16, 17]. Little literature devoted to the effect of biased technical progress on China’s manufacturing employment. What’s the characteristics of the technical progress bias in China’s manufacturing industries, and whether it have a broad impact on employment? To fill this gap, we conduct a special empirical study based on the panel data of 28 manufacturing industries from 1999 to 2010 of China. The results indicate the technical progress of China’s manufacturing is skill biased and has a significant positive impact on the total employment and unskilled labor’s employment but insignificant impact on the skilled labor’s employment due to threshold effect.

## 2. Measurement of technical progress bias

### 2.1 Research model

In this section, we use the stochastic frontier model based on transcendental logarithm function to measure the bias of technical progress of China’s manufacturing industry. The general form of stochastic frontier model is:

$$\ln Y_{it}=\ln f(x_{it},\beta )+v_{it}-u_{it}$$ (1)

where, *Y*_*it*_ represents the actual output, *f*(*x*_*it*_,*β*) represents the output of the enterprise on the production possibility frontier, i.e. the output when the enterprise has no efficiency loss, *x*_*it*_ and *β* represents the input vector of production factors and the coefficient vector of production factors respectively, *v*_*it*_ represents the random shocks, assuming *v*_*it*_ obeys the standard normal distribution, i.e $v_{it}∼N(0,\sigma_{v}^{2})$. *u*_*it*_ is technical inefficiency term, which reflects the efficiency loss of enterprise during production, *u*_*it*_ and *v*_*it*_ is assumed to be independent with each other.

Base on the setting of Battese and Coelli (1992) [18], *u*_*it*_ can be expressed as follows:

$$u_{it}=u_{i}\exp[-\eta (t-T)]$$ (2)

Where *T* is the time interval of enterprise production, *u*_i_ subject to truncated normal distribution, that is $u_{i}∼N^{+}(\mu ,\sigma_{u}^{2})$. The meaning of this setting is assuming the production of most enterprises are between completely efficient and completely inefficient, only a few enterprises are in the extreme situation.*η* is the change rate of *u*_*it*_. Eq (2) shows that *u*_*it*_ decreases with the increase of time *t*. In order to measure the bias of technical progress between skilled labor and unskilled labor, the production function should contain these three production factors: capital, skilled labor and unskilled labor. The transcendental logarithm function including these three factors is established as follows:

$$\ln f(x_{it},\beta )=\beta_{0}+\beta_{T}T+\beta_{K}\ln K_{it}+\beta_{H}\ln H_{it}+\beta_{L}\ln L_{it}+\beta_{TT}T^{2}/2+$$

$$\beta_{KK}{\left(\ln K_{it}\right)}^{2}/2+\beta_{HH}{\left(\ln H_{it}\right)}^{2}/2+\beta_{LL}{\left(\ln L_{it}\right)}^{2}/2+\beta_{TK}T\ln K_{it}+\beta_{TH}T\ln H_{it}$$

$$+\beta_{TL}T\ln L_{it}+\beta_{KH}\ln K_{it}\ln H_{it}+\beta_{KL}\ln K_{it}\ln L_{it}+\beta_{HL}\ln H_{it}\ln L_{it}$$ (3)

where, *K*_*it*_、*H*_*it*_、*L*_*it*_ represent capital input, skilled labor input and unskilled labor input respectively. *T* is the time trend term. Substitute Eq (3) into Eq (1), we can get the stochastic frontier model based on transcendental logarithm function as bellow:

$$\ln Y_{it}=\beta_{0}+\beta_{T}T+\beta_{K}\ln K_{it}+\beta_{H}\ln H_{it}+\beta_{L}\ln L_{it}+\beta_{TT}T^{2}/2+\beta_{KK}{\left(\ln K_{it}\right)}^{2}/2$$

$$+\beta_{HH}{\left(\ln H_{it}\right)}^{2}/2+\beta_{LL}{\left(\ln L_{it}\right)}^{2}/2+\beta_{TK}T\ln K_{it}+\beta_{TH}T\ln H_{it}+\beta_{TL}T\ln L_{it}$$

$$+\beta_{KH}\ln K_{it}\ln H_{it}+\beta_{KL}\ln K_{it}\ln L_{it}+\beta_{HL}\ln H_{it}\ln L_{it}+v_{it}-u_{it}$$ (4)

Since the disturbance term has violated the classical hypothesis, we will use the maximum likelihood estimation method (MLE) to estimate it and calculate the technical progress bias. According to Formula (4), the output elasticity of each factor can be calculated as follows:

$$\eta_{Hit}=\partial \ln Y_{it}/\partial \ln H_{it}=\beta_{H}+\beta_{HH}\ln H_{it}+\beta_{KH}\ln K_{it}+\beta_{HL}\ln L_{it}+\beta_{TH}T$$ (5)

$$\eta_{Lit}=\partial \ln Y_{it}/\partial \ln L_{it}=\beta_{L}+\beta_{LL}\ln L_{it}+\beta_{KL}\ln K_{it}+\beta_{HL}\ln H_{it}+\beta_{TL}T$$ (6)

Where *η*_*Hit*_ and *η*_*Lit*_ represent the output elasticity of skilled labor and unskilled labor respectively. According to the calculation formula of biased technical progress index designed by Diamond (1965) [19], combining Eqs (4), (5) and (6), the bias of technical progress between skilled labor and unskilled labor can be calculated as:

$$DBias_{HL}=\partial \ln MPH/\partial T-\partial \ln MPL/\partial T=\beta_{TH}/\eta_{Hit}-\beta_{TL}/\eta_{Lit}$$ (7)

Where *DBias*_*HL*_ is the biased technical progress index, standing for the technical progress bias between skilled and unskilled labor. MPH and MPL represent the marginal output of skilled and unskilled labor respectively. When *Dbias*_*HL*_>0,the growth rate of marginal output of skilled labor caused by technical progress was larger than that of the unskilled labor. At this moment, technical progress is tend to skilled labor and the higher *DBias*_*HL*_ is, the more of technical progress tend to skilled labor. On the contrary, when *Dbias*_*HL*_<0,technical progress tends to unskilled labor and the smaller *DBias*_*HL*_ is, the more of technical progress tend to unskilled labor.

### 2.2 Data description

We use the panel data of different sub-industries of the manufacturing to calculate the biased technical progress index. The data caliber is the enterprises above the scale. The missing data of individual years are supplemented by interpolation method. Since 2012, the National Bureau of Statistics has adjusted the standards for enterprises above the scale, so the time interval is 1999–2011. We eliminate the arts and crafts, waste gas resources and other manufacturing industries due to the lack of data. The data sources and processing methods of each variable are as follows:

- Output Y is measured by industrial added value of various manufacturing industries, the unit is 100 million RMB. The data comes from China statistical yearbook.
- Capital input K is calculated by the perpetual inventory method (PIM); the unit is 100 million RMB. The calculation method is referred to Chen et al. [20]. In addition, there was no data of enterprises above the scale before 1998, therefore, the data were obtained by using the mean annual rate of change according to the relevant data from 1998 to 2011.
- Skilled labor H is represented by the indicators of technical activity involved persons [21]. The data of technical activists came from China science and technology statistics yearbook.
- Unskilled labor force L is defined as employees other than those engaged in technical activities. Therefore, non-skilled employment can be obtained by subtracting skilled employment from the average number of employees. The descriptive statistics of each variable are given in Table 1A of S1 Appendix.

Furthermore, it should be noted that this article does not contain any studies with human participants or animals performed by any of the authors.

### 2.3 Estimation results and testing

The estimation method is Maximum likelihood estimation method (MLE). The results of parameters estimated are reported in Table 1.

**Table 1:** Estimation results of the stochastic frontier model.

Variable	Coefficient	Variable	Coefficient
Constant	154.9789**(75.9091)	lnHlnL	-0.0411(0.0450)
T	4.6020***(1.5550)	TlnK	-0.0278***(0.0092)
lnK	0.1121(0.4713)	TlnH	0.0038(0.0059)
lnH	-0.3405(0.2974)	TlnL	0.1924***(0.0061)
lnL	2.2714***(0.5021)	σ_v_^2^	0.0085***(0.0007)
T^2^/2	0.1470***(0.0352)	σ_u_^2^	0.3289***(0.0988)
(lnK)^2^/2	0.1646(0.1059)	σ^2^	0.3375***(0.0988)
(lnH)^2^/2	-0.1030**(0.0461)	Γ	0.9747***(0.0077)
(lnL)^2^/2	-0.1283(0.1045)	μ	228.9419**(98.2856)
lnKlnH	0.1497***(0.0434)	η	-0.0281***(0.0039)
lnKlnL	-0.1713**(0.0796)	Lnf(.)	268.0659[0.0000]

Notes: Levels of significance: ***1%, **5%, *10%. The value in parentheses are standard error, the value in brackets are P value.

As shown in Table 1, most of the estimated coefficients are significant, indicating that the model used can simulate the actual production situation of the manufacturing very well. γ represents the proportion of inefficiency variance in the total variance, that is $\gamma =\sigma_{u}^{2}/(\sigma_{u}^{2}+\sigma_{v}^{2})$, the closer of γ is to 1, the more likely it is that the actual output of the enterprise does not reach the production possibility boundary is mainly caused by technical inefficiency. As can be seen from Table 2, the estimated value of γ is 0.9747, which is very close to 1 and significant at the 1% level, indicating that it is reasonable to add technical inefficiency into the model. Next, we will test the model from the following three aspects:

The first is to test the rationality of the transcendental logarithm function; If the original hypothesis *H*_0_: *β*_*T*_ = *β*_*TT*_ = *β*_*KK*_ $=\beta_{HH}=\beta_{LL}=\beta_{TK}=\beta_{TH}=\beta_{TL}=\beta_{KH}=\beta_{KL}=\beta_{HL}=0$ is true, the production function should be set in the form of Cobb-douglas; otherwise, the transcendental logarithm function is reasonable.

The second is to test whether there is technical progress in the production function. In the transcendental logarithm function, the technical progress is represented by the time trend term *T*. If the original hypothesis $H_{0}:\beta_{T}=\beta_{TT}=\beta_{TK}=\beta_{TH}=\beta_{TL}=0$ is true, it indicates that there is no technical progress in the production function; otherwise, the technical progress is exist.

The third is to test if technical progress bias exist in the production function. The technical progress bias in the transcendental logarithm function is expressed by the interaction term of time trend term T and production factors. If the original hypothesis $H_{0}:\beta_{TK}=\beta_{TH}=\beta_{TL}=0$ is true, it indicates that there is no technical progress bias, and vice versa.

The three test above adopts the generalized likelihood ratio test, in which the generalized likelihood ratio statistic is set as $LR=-2\ln[L(H_{0})/L(H_{1})]$, obeys mixed *X*^2^ distribution, *L*(*H*_0_) and *L*(*H*_1_) are the likelihood function value under the original hypothesis and the alternative hypothesis respectively. The inspection results are summarized in Table 2.

**Table 2:** Test results of model setting.

Original hypothesis	LR	P	Test Result
$\beta_{T}=\beta_{TT}=\beta_{KK}=\beta_{HH}=\beta_{LL}=\beta_{TK}=\beta_{TH}$ = *β*_*TL*_ *=β*_*KH*_ *=β*_*KL*_ = *β*_*HL*_ = 0	206.35	0.00	Refused
*β*_*T*_ = *β*_*TT*_ *β*_*TK*_ = *β*_*TH*_ = *β*_*TL*_ = 0	109.77	0.00	Refused
*β*_*TK*_ = *β*_*TH*_ = *β*_*TL*_ = 0	33.71	0.00	Refused

It can be seen from the second line of Table 2 that the generalized likelihood ratio test rejects the Cobb-Douglas production function null hypothesis, indicating that the transcendental logarithm function is reasonable. As shown in the third row of Table 3, the generalized likelihood ratio test rejects the assumption that there is no technical progress in the production function, indicating that technical progress does exist in manufacturing industry. The fourth line of Table 2 says that the generalized likelihood ratio test rejects the null hypothesis that there is no technical progress bias in the production function, indicating that the technical progress in the manufacturing industry is not neutral, but shows the feature of factor bias. Sum up, the transcendental logarithm function can reflect the actual situation of manufacturing better, and there is efficiency loss in manufacturing production, so it is necessary to add technical inefficiency item to build stochastic frontier model. Therefore, it is reasonable to use the stochastic frontier model based on the transcendental logarithm function to simulate the manufacturing production.

**Table 3:** Baseline regression results of the impact on employment.

lnL			lnS		lnUS	
	Fixed Effect	Random Effect	Fixed Effect	Random Effect	Fixed Effect	Random Effect
DBias	0.0067** (0.0031)	0.0078** (0.0036)	-0.0102 (0.0067)	-0.0136* (0.0082)	0.0072** (0.0032)	0.0082** (0.0037)
lnW	-0.6317*** (0.0461)	-0.6742*** (0.0478)				
lnWS			-0.4715*** (0.0517)	-0.8131*** (0.0493)		
lnWUS					-0.6138*** (0.0474)	-0.6421*** (0.0491)
lnY	0.3538*** (0.0350)	0.4055*** (0.0392)	0.2552*** (0.0738)	0.0256 (0.0677)	0.3480*** (0.0363)	0.3988*** (0.0403)
lnK	0.8511*** (0.0495)	0.6525*** (0.0497)	-0.2442** (0.1087)	0.0699 (0.0738)	0.8928*** (0.0515)	0.6897*** (0.0515)
lnIM	0.0342** (0.0149)	0.0436** (0.0160)	0.0302 (0.0335)	0.1299*** (0.0276)	0.0312** (0.0154)	0.0389** (0.0165)
lnEX	-0.0001 (0.0177)	0.0491** (0.0190)	0.0758* (0.0394)	0.0626** (0.0298)	-0.0033 (0.0184)	0.0457** (0.0197)
lnRD	-0.0794*** (0.0245)	-0.1043*** (0.0269)	0.3067*** (0.0526)	0.7049*** (0.0504)	-0.1030*** (0.0253)	-0.1333*** (0.0277)
lnFDI	-0.1706*** (0.0312)	-0.0857** (0.0328)	-0.0792 (0.0689)	-0.1584*** (0.0477)	-0.1642*** (0.0324)	-0.0775** (0.0340)
Constant	1.3580*** (0.1749)	1.8244*** (0.1775)	3.5105*** (0.3697)	1.6942*** (0.2511)	1.1105*** (0.1824)	1.6072*** (0.1849)
Observations	364	364	364	364	364	364
Hausman Test	109.09 [0.0000]		130.08 [0.0000]		106.03 [0.0000]	

Notes: Levels of significance: *** 1%

** 5%

* 10%. The value in parentheses are the standard error, and the value in brackets are the P value.

### 2.4 The Preference of technical progress bias in China’s manufacturing

The biased technical progress index can be calculated based on Eqs (5), (6) and (7) combing the parameter estimate in Table 1. The technical progress bias of 28 sub-industries of China’s manufacturing are listed in Table 4A of S1 Appendix. In order to avoid the interference of some extreme values on the symbol of the technical progress bias, the values are indented at the level of 1% quantile. Table 4A of S1 Appendix shows, the technical progress bias of 16 industries were toward the skilled labor in the sample period, while the remaining 12 industries were toward the unskilled labor. So for most of China’s manufacturing industries, the technical progress bias toward the skilled labor.

## 3. Empirical test of the impact on employment

### 3.1. Model and variable description

For empirical analysis, we construct the static panel model as follows:

$$\ln L_{it}=\gamma_{0}+\gamma_{1}DBias_{it}+\gamma_{2}\ln W_{it}+\gamma_{3}\ln Y_{it}+\gamma_{4}\ln K_{it}+\gamma_{5}\ln IM_{it}$$

$$+\gamma_{6}\ln EX_{it}+\gamma_{7}\ln RD_{it}+\gamma_{8}\ln FDI_{it}+\varepsilon_{it}$$ (8)

$$\ln S_{it}=\alpha_{0}+\alpha_{1}DBias_{it}+\alpha_{2}\ln WS_{it}+\alpha_{3}\ln Y_{it}+\alpha_{4}\ln K_{it}+\alpha_{5}\ln IM_{it}$$

$$+\alpha_{6}\ln EX_{it}+\alpha_{7}\ln RD_{it}+\alpha_{8}\ln FDI_{it}+\varepsilon_{it}$$ (9)

$$\ln US_{it}=\beta_{0}+\beta_{1}DBias_{it}+\beta_{2}\ln WUS_{it}+\beta_{3}\ln Y_{it}+\beta_{4}\ln K_{it}+\beta_{5}\ln IM_{it}$$

$$+\beta_{6}\ln EX_{it}+\beta_{7}\ln RD_{it}+\beta_{8}\ln FDI_{it}+\varepsilon_{it}$$ (10)

Where i represents different industries, t represents the year, the range is 1999–2011; *ε*_*it*_ is the random disturbance term. The explained variables are L, S and US, represent the total employment, skilled employment and non-skilled employment respectively. The measurement method is the same as the previous. The core explanatory variable is DBias measured in the previous section.

The rest is a set of control variables:

- W represents the actual average labor salary in different industries, the unit is 1000 RMB;
- WS represents the actual wage of skilled labor in different industries, the unit is 1000 RMB;
- WUS represents the actual wage of unskilled labor, the unit is 1000 RMB;
- Y represents the output of different industries, the same way as the previous section.
- K represents the capital stock of, the same way as the previous section.
- IM and EX represent the import and export respectively; the unit is 100 million RMB.
- RD represents the R&D capital stock; the unit is 100 million RMB.
- FDI on behalf of the foreign direct investment, the unit is 100 million RMB, using the perpetual inventory method (PIM), the detailed data processing method is similar to the calculation of the capital stock, the only difference is the basic data, change to the original value of foreign fixed assets and the net value of foreign fixed assets, the data is from China industrial economic statistical yearbook. In addition, except for the technical progress bias index, the other variables are logarithm in the model. Table 5A of S1 Appendix shows the descriptive statistics of the above variables.

### 3.2 Baseline regression

We estimated Eqs (8), (9) and (10) as fixed effect models and random effect models respectively to choose one model with Hausman test. The fixed effects model is estimated by LSDV method, and the random effects model is estimated by GLS method. The results of parameter estimation are reported in Table 3.

The last line of Table 3 shows that the Hausman test values of the three models are 109.09, 130.08 and 106.03 respectively, indicating that the three models should choose the fixed-effect model. According to the second column, when technical progress towards skilled labor, the total employment of manufacturing industry will increase. Specifically, if technical progress towards skilled labor for one unit, the total employment of manufacturing industry will increase about 0.67%, and the result is significant at 5% level. It can be seen from column 4, that, during the sample period, the impact of technical progress on the skilled labor’s employment is not significant, indicating that the transmission channel of technical progress skill bias on skilled labor’s employment is disturbed by some factors, making the influence effect insignificant. According to column 6, when technology favors skilled labor, the employment of unskilled labor in the manufacturing industry will increase. Specifically, the employment of unskilled labor in the manufacturing industry will increase by about 0.72% if technical progress favors skilled labor by 1 unit, and the result is significant at the significance level of 5%.

### 3.3 Endogenous treatment

Technical progress bias can affect the total employment of manufacturing, on the contrary, technical progress bias is influenced by labor endowment [3, 10]. Therefore, there may be a bidirectional causality relationship between technical progress bias and total manufacturing employment. This endogenous problem may cause the estimation error of baseline regression. Thus, we select the technical progress bias with one lag period as the instrumental variable to estimate the model through 2SLS method. The results are shown in Table 4.

**Table 4:** The estimation result of IV-2sls.

	lnL		lnS		lnUS
DBias	0.0233*(0.0127)		0.0097(0.0285)		0.0244*(0.0131)
lnW	-0.6751***(0.0574)				
lnWS			-0.5899***(0.0670)		
lnWUS					-0.6663***(0.0597)
lnY	0.3822***(0.0462)		0.2962***(0.0892)		0.3823***(0.0479)
lnK	0.8067***(0.0557)		-0.2516*(0.1312)		0.8431***(0.0573)
lnIM	0.0362**(0.0161)		0.5538(0.0376)		0.0341**(0.0166)
lnEX	0.0076(0.0188)		0.0703*(0.0425)		0.0055(0.0194)
lnRD	-0.0569**(0.0282)		0.3229***(0.0610)		-0.0791***(0.0289)
lnFDI	-0.1705***(0.0333)		-0.0809(0.0744)		-0.1622***(0.0343)
Observations	336		336		336
Anderson canon.corr.LM	19.894[0.0000]		19.600[0.0000]		19.867[0.0000]
Cragg-Donald Wald F	20.715		20.389		20.685
The critical value of Stock-Yogo weak IV test		10% errors		16.38	
		15% errors		8.96	
		20% errors		6.66	
		25% errors		5.53	

Notes: Levels of significance: ***1%

**5%

*10%. The value in parentheses are the standard error, and the value in brackets are the P value.

As can be seen from Table 4, the Anderson Canon. Corr. LM statistics of the three models were 19.894, 19.6 and 19.867 respectively, all of which rejected the null hypothesis at the significance level of 1%, indicating that there was no "unrecognizable" problem in the selected instrumental variables. Cragg-donald Wald F statistics of the three models were 20.715, 20.3892 and 20.685, respectively, larger than the critical value of stock-yogo weak IV test under 10% bias, indicating that the selected instrumental variables did not have the problem of "weak instrumental variables". Summing up, the instrumental variables we selected are valid. It can be seen from column 2 that technical progress tends to skilled labor, which will increase the total employment of manufacturing industry, and the result is significant at the significance level of 10%. It can be seen from the third column that, after dealing with endogenous problems, the impact of technical progress on the employment of skilled workers in manufacturing industry is still not significant. It can be seen from column 4 that when technical progress favors skilled labor, employment of unskilled labor in the manufacturing industry will increase. Specifically, if technical progress favors skilled labor by 1 unit, employment of unskilled labor in the manufacturing industry will increase by about 2.4%, and the result is significant at the 10% significance level. This indicates that the possible endogenous problems in the model do not affect our basic conclusion.

### 3.4 Robustness checks

The robustness of the model will be tested in two ways: one is to change part of the control variables, and the other is to change the time interval.

#### 3.4.1 Replace control variables

In the estimation of baseline regression and instrumental variables, we use the perpetual inventory method (PIM) to calculate the capital stock and foreign direct investment. In some studies, net fixed assets and net foreign fixed assets are also used to measure capital stock and foreign direct investment [22–24]. Therefore, we use net fixed assets to measure the capital stock, net foreign fixed assets to measure foreign direct investment, and the technical progress bias index with one period lagged as the instrumental variable to re-estimate the model by using the two-stage least square method (2SLS). The parameter estimation results are shown in Table 5.

**Table 5:** Robustness check I: Changing part of the control variables.

	lnL	lnS		lnUS
DBias	0.0240* (0.0127)	0.0057 (0.0279)		0.0255* (0.0132)
lnW	-0.6487*** (0.0609)			
lnS		-0.5865*** (0.0678)		
lnUS				-0.6306*** (0.0638)
lnY	0.3775*** (0.0464)	0.2821*** (0.0869)		0.3770*** (0.0486)
lnK	0.7610*** (0.0527)	-0.2608** (0.1217)		0.7928*** (0.0546)
lnIM	0.0407** (0.0166)	0.0406 (0.0381)		0.0393** (0.0172)
lnEX	0.0156 (0.0195)	0.0486 (0.0433)		0.0143 (0.0203)
lnRD	-0.0639** (0.0286)	0.2975*** (0.0604)		-0.0858*** (0.0296)
lnFDI	-0.1736*** (0.0299)	0.0209 (0.0652)		-0.1701*** (0.0311)
Observations	336	336		336
Anderson canon.corr. LM	20.521 [0.0000]	20.318 [0.0000]		20.464 [0.0000]
Cragg-Donald Wald F	21.415	21.188		21.351
The critical value of Stock-Yogo weak IV test	10% errors		16.38	
	15% errors		8.96	
	20% errors		6.66	
	25% errors		5.53	

Notes: Levels of significance: *** 1%

** 5%

* 10%. The value in parentheses are the standard error, and the value in brackets are the P value.

According to the Anderson Canon. Corr. LM statistics and Cragg-Donald Wald F statistics of the three models in Table 5, the selection of instrumental variables is effective. It can be seen from column 2 that technical progress tends to skilled labor, which will increase the total employment of manufacturing industry, and the result is significant at the significance level of 10%. It can be seen from the third column that the impact of technical progress bias on the employment of skilled workers in manufacturing industry was still not significant. It can be seen from column 4 that when technical progress favors skilled labor, employment of unskilled labor in the manufacturing industry will increase. Specifically, when technical progress favors skilled labor for one unit, employment of unskilled labor in the manufacturing industry will increase by about 2.6%, and the result is significant at 10% significance level. The results are robust.

#### 3.4.2 Change the time interval

In the baseline regression, our data time interval was 1999–2011. We changed the data time interval to 2001–2011. The two-stage least square method (2SLS) was still used to re-estimate the model, the parameter estimation results were shown in Table 6.

**Table 6:** Robustness checks Ⅱ: Changing time interval.

	lnL	lnS		lnUS
DBias	0.0191** (0.0095)	-0.0038 (0.0244)		0.0200** (0.0098)
lnW	-0.6413*** (0.0578)			
lnWS		-0.0038 (0.0244)		0.0200** (0.0098)
lnWUS				-0.6164*** (0.0592)
lnY	0.3298*** (0.0442)	0.2796*** (0.1022)		0.3265*** (0.0457)
lnK	0.7504*** (0.0591)	-0.3358** (0.1594)		0.7754*** (0.0608)
lnIM	0.0366** (0.0176)	0.0919** (0.0469)		0.0345* (0.0181)
lnEX	0.0214 (0.0206)	0.1512*** (0.0535)		0.0136 (0.0212)
lnRD	0.0251 (0.0328)	0.3746*** (0.0836)		0.0015 (0.0336)
lnFDI	-0.1805*** (0.0392)	-0.0969 (0.0990)		-0.1626*** (0.0402)
Observations	280	280		280
Anderson canon.corr.LM	23.862 [0.0000]	24.490 [0.0000]		23.768 [0.0000]
Cragg-Donald Wald F	25.521	26.265		25.410
The critical value of Stock-Yogo weak IV test	10% errors		16.38	
	15% errors		8.96	
	20% errors		6.66	
	25% errors		5.53	

Notes: Levels of significance: *** 1%

** 5%

* 10%. The value in parentheses are the standard error, and the value in brackets are the P value.

According to the Anderson Canon. Corr. LM statistics and Cragg-Donald Wald F statistics of the three models in Table 6, the selection of instrumental variables is effective. It can be seen from column 2 that technical progress tends to skilled labor, which will increase the total employment of manufacturing industry, and the result is significant at the significance level of 10%. It can be seen from the third that, after dealing with endogenous problems, the impact of technical progress bias on the employment of skilled workers in manufacturing industry is still not significant. It can be seen from column 4 that when technical progress favors skilled labor, unskilled labor employment in manufacturing industry will increase. Specifically, technical progress favors skilled labor by 1 unit, and employment of non-skilled labor in manufacturing industry will increase by about 2%, and the result is significant at 10% significance level. This indicates that change the time interval do not affect the results.

### 3.4 Discussion

The empirical results above indicate that for the employment of the skilled labor in manufacturing, the impact of the skilled biased technical progress is not significant. What’s the reason? A possible explanation is a threshold effect exist. China’s manufacturing may not pass this "threshold" during the sample period, making the impact insignificant. When the marginal outputs of the skilled labor increase due to skill biased technical progress, not all companies will increase the demand for skilled labor, there are two types of companies who are more likely to hire more skilled labor: one is the ones with high level of development to bear the failure risk in R&D, the others are the ones have certain level of R&D accumulation so that the benefits to hire skilled labor outweigh the costs. For normal expectations, when skill-biased technological progress occurs, the marginal output of skilled labor will increase, and manufacturing enterprises’ demand for skilled labor will increase accordingly. As a result, the wage of skilled labor will rise, leading to more skilled labor entering the manufacturing industry. Under the new equilibrium state, the employment of skilled labor in the manufacturing industry will increase. However, this transmission mechanism is only applicable to enterprises with a certain level of development and a certain accumulation of R&D investment. For small and medium-sized enterprises, due to the existence of R&D risks, the above-mentioned transmission mechanism is disturbed, resulting in a slow growth in the demand for skilled labor under the impact of skills-biased technological progress, so the employment growth of skilled labor is not significant. On the contrary, during the sample period, skilled labor and unskilled labor in China’s manufacturing industry show a substitution relationship. When skills-enhanced technological progress occurs, the marginal output of unskilled labor also increases, leading to an increase in the employment demand of unskilled labor, and thus an increase in the employment of unskilled labor and the increase in total employment. Based on these two reasons, we think the impact the skill biased technical progress on the employment in manufacturing may have two threshold effects: one is the threshold effect of the development level; the other is the threshold effect of the R&D accumulation level. For China’s manufacturing, the development level and R&D accumulation level of most industries may not pass the threshold, therefor, the employment of the skilled labor in China’s manufacturing is not significantly activated by the skill biased technical progress. We will test these two threshold effect next.

## 4. Threshold effect test

The verification of threshold effect is divided into three steps. The first is to test whether threshold effect exists, the second is to calculate the threshold value, the third is to calculate the mean value of the threshold variables and compare them with the threshold values to find their interval. In this paper, two threshold variables are selected, industry value-added per capita and R&D capital stock. The former measures the development level of the industry, while the latter measures the R&D accumulation in the industry. The test method is the panel threshold model proposed by Hansen [25]. The threshold variable should be exogenous. Since the threshold regression method is used to estimate the model after ranking the threshold variables, if the threshold variable contains a strong time trend, this trend will be brought into the model, and the existence of the trend will change the likelihood distribution test of mutation points. More importantly, in this case, the confidence interval cannot be constructed, making the problem impossible to study. Therefore, when selecting threshold variables, try to avoid choosing absolute indicators with trends, but should choose exogenous variables.

### 4.1 Model and variable description

Referring to Hansen (1999), the model was constructed as follows:

$$\ln S_{it}=\beta_{1}DBias_{it}\times I(Q_{it}<\gamma_{1})+\sum_{k=2}^{n}\beta_{k}DBias_{it}\times I(\gamma_{k-1}<Q_{it}<\gamma_{k})+$$

$$\beta_{n+1}DBias_{it}\times I(Q_{it}>\gamma_{n})+\alpha \ln X_{it}+\lambda_{i}+\varepsilon_{it}$$ (11)

Where i represents different manufacturing industries.t represents the year, 1999–2011; λ is the individual effect term, ε is the random disturbance term. I(·) is an indicator function. If the condition in parentheses is true, then I(·) = 1;On the contrary, I(·) = 0. With the introduction of indicator function, we can easily describe the different effects of technical progress bias on the employment of skilled workers in different threshold intervals. For example, if *Q*_*it*_<*γ*_1_, *I*(*Q*_*it*_<*γ*_1_) = 1, the remaining indicator functions are all 0,the elasticity coefficient of the impact of technical progress bias on skilled labor employment isβ_1_. If *Q*_*it*_>*γ*_*n*_, *I*(*Q*_*it*_>*γ*_*n*_) = 1, the elasticity coefficient becomes β_n+1_.

The explained variable S_it_ stands for skilled labor in manufacturing. The core explanatory variable is DBias. The threshold variable is Q, which stands for the industry value-added per capita and the R&D capital stock respectively. γ is the threshold value. In model (11), we set the existence of n thresholds. In the following text, we will determine the number of thresholds and estimate their values. X is a set of control variables, including output Y, capital stock K, import IM, export EX, R&D capital stock and foreign investment FDI.

### 4.2 Empirical results based on industry value-added per capita

We use the industry value-added as threshold first. The null hypothesis was threshold effect does not exist. According to the recommendation of Hansen (1999), we adopted the likelihood ratio test and constructed the statistic as $F=[SSR*-SSR(\overset{\wedge }{\gamma })]/\overset{\wedge }{\sigma^{2}}$, where SSR* represents the sum of residual squares under the original hypothesis, $SSR(\overset{\wedge }{\gamma })$ represents the sum of residual squares under threshold effect, and $\overset{\wedge }{\sigma^{2}}=SSR(\overset{\wedge }{\gamma })/n(T-1)$ is a consistent estimate of the variance of the random disturbance term. Because the threshold value γ is unrecognizable under the null hypothesis, causing the large sample distribution of $[SSR*-SSR(\overset{\wedge }{\gamma })]/\overset{\wedge }{\sigma^{2}}$ do not obey the *X*^2^ distribution, and can’t get the critical value of the distribution. Therefore, we use bootstrap method to calculate the critical value. After the single threshold effect test, we will further test the double threshold effect and triple threshold effect, where the null hypothesis of the double threshold effect test is there is a single threshold, while the null hypothesis of the triple threshold effect test is there is a double threshold. The test results are shown in Table 7A of S1 Appendix.

See Table 7A of S1 Appendix, we reject the null hypothesis that there is no threshold effect at the significance level of 5% base on single threshold effect, accepting the alternative hypothesis that there is a single threshold effect. Double threshold effect and triple threshold effect did not pass. Then, model (11) can be written as:

$$\ln S_{it}=\beta_{1}DBias_{it}\times I(RY_{it}<\gamma )+\beta_{2}DBias_{it}\times I(RY_{it}>\gamma )+\alpha \ln X_{it}+\lambda_{i}+\varepsilon_{it}$$ (12)

**Fig 1:**
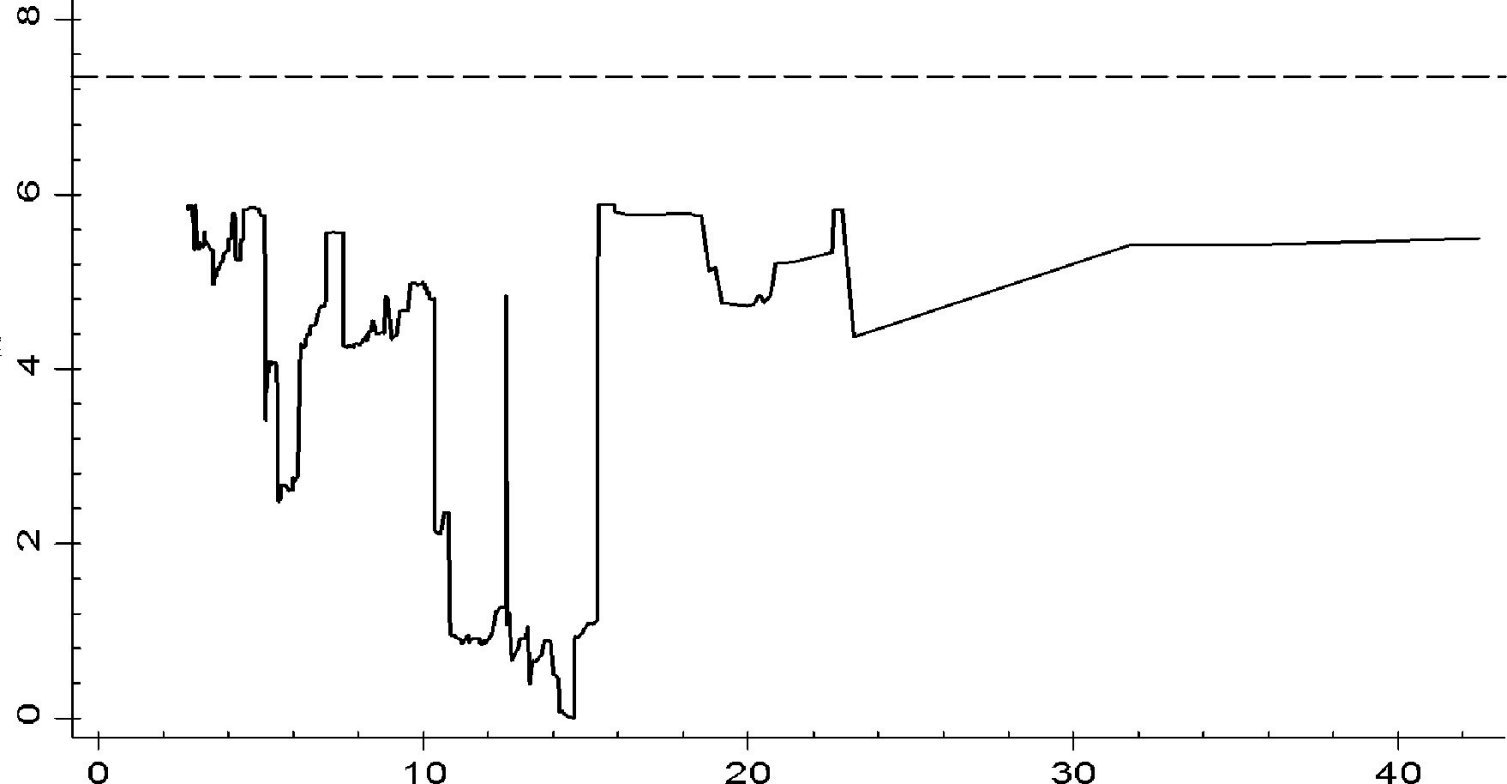
Visualization of threshold estimation test results.

Now we estimate the model (12). According to Hansen (1999), the first step is convert model (12) into deviation form to remove individual effect *λ*_*i*_, then, for any given threshold value *γ*, use OLS method to estimate the parameters and calculate the sum of squared residuals *SSR*(*γ*). Finally, select *γ* which minimizes *SSR*(*γ*) as the estimated threshold value. Hansen (1999) required that explanatory variables must be unrelated to the random disturbance terms, otherwise the estimation results will be biased. So, we estimated the explanatory variable with one period lagged. When the threshold estimation was obtained, we still need to test its authenticity. According to Hansen [25], the original hypothesis for threshold estimation is the threshold estimate equal to the real value. The statistics value is the likelihood-ratio statistic $LR=[SSR(\gamma )-SSR(\overset{\wedge }{\gamma })]/\overset{\wedge }{\sigma^{2}}$, where *SSR*(*γ*) is the sum of residual squares under the null hypothesis, $SSR(\overset{\wedge }{\gamma })$ is the sum of residual squares under the alternative hypothesis-said, $\overset{\wedge }{\sigma^{2}}=SSR(\overset{\wedge }{\gamma })/n(T-1)$ is a consistent estimate of the variance of the random disturbance. Fig 1 shows the test result of threshold estimation. Table 7 shows the results of parameter estimation.

**Table 7:** Threshold value and interval.

	lnS(industry value-added)		lnS (R&D capital stock)	
	Coefficient	95%Confidence Interval	Coefficient	95%Confidence Interval
DBias×I(RY<γ)	-0.010(0.009)	[-0.028, 0.008]	-0.013(0.009)	[-0.031, 0.005]
DBias×I(RY>γ)	0.048**(0.024)	[0.001, 0.095]	0.045**(0.020)	[0.007, 0.084]
γ	14.633	[2.749, 42.467]	276.816	[150.785, 2214.876]

Notes: Levels of significance: ***1%

**5%

*10%. The value in parentheses are standard error of estimation coefficient

See Fig 1, when the industry value-added per capita is taken as the threshold variable, the LR value of the threshold estimate is far less than the critical value 7.35 at the significance level of 5%. Therefore, the null hypothesis is accepted, indicating the threshold estimate is true and effective. According to Table 7, when the industry value-added per capita is taken as the threshold variable, the estimated threshold value is 14.633, meaning when the industry value-added per capita is less than 14.633, the impact of skill biased technical progress on the employment of skilled labor is not significant, otherwise, it becomes significant. We take Chow test to verify the significance level of the threshold. Ho: no Structural Change,the Chow Test is 4.37,P-Value > F(2, 234) = 0.021, which means the threshold is significant. Specifically, technical progress is biased towards skilled labor for 1 unit, the employment of skilled labor will increase by about 4.8% at the significance level of 5%. Further, we calculated the average value of industry value-added per capita in manufacturing in the sample period, and classified the manufacturing industries according to the threshold estimate. The results show that there are only two industries exceeded the threshold estimate value during the sample period, they are Tobacco Industry and Communication equipment, computer and other electronic equipment manufacturing Industry, the remaining 26 industries are lower than the threshold estimate. The results show that the development level of most industries in the sample period is still insufficient.

### 4.3 Empirical results based on R&D capital stock

Next, we take R&D capital stock as the threshold to estimate and check. The results of the threshold effect test were shown in Table 8.

**Table 8:** Threshold effect test results.

Model	F Value	P Value	Sample	1%	5%	10%
Single threshold	8.687*	0.070	300	14.814	11.555	7.817
Double threshold	3.686	0.183	300	14.916	8.560	6.956
Triple threshold	2.434	0.220	300	9.539	5.709	4.113

Notes: Levels of significance: ***1%, **5%

*10%. The P value and critical values were obtained by repeated sampling for 300 times.

See Table 8, we reject the null hypothesis that there is no threshold effect at the significance level of 10% in single threshold effect test, accepting the alternative hypothesis that there is a single threshold effect. Double threshold effect and triple threshold effect did not pass. Therefore, there is also only a single threshold effect about the impact of the skill biased technical progress on the employment of skilled labor in manufacturing when the R&D capital stock is taken as the threshold variable. Then, model (11) can be written as:

$$\ln S_{it}=\beta_{1}DBias_{it}\times I(RD_{it}<\gamma )+\beta_{2}DBias_{it}\times I(RD_{it}>\gamma )+\alpha \ln X_{it}+\lambda_{i}+\varepsilon_{it}$$ (13)

**Fig 2:**
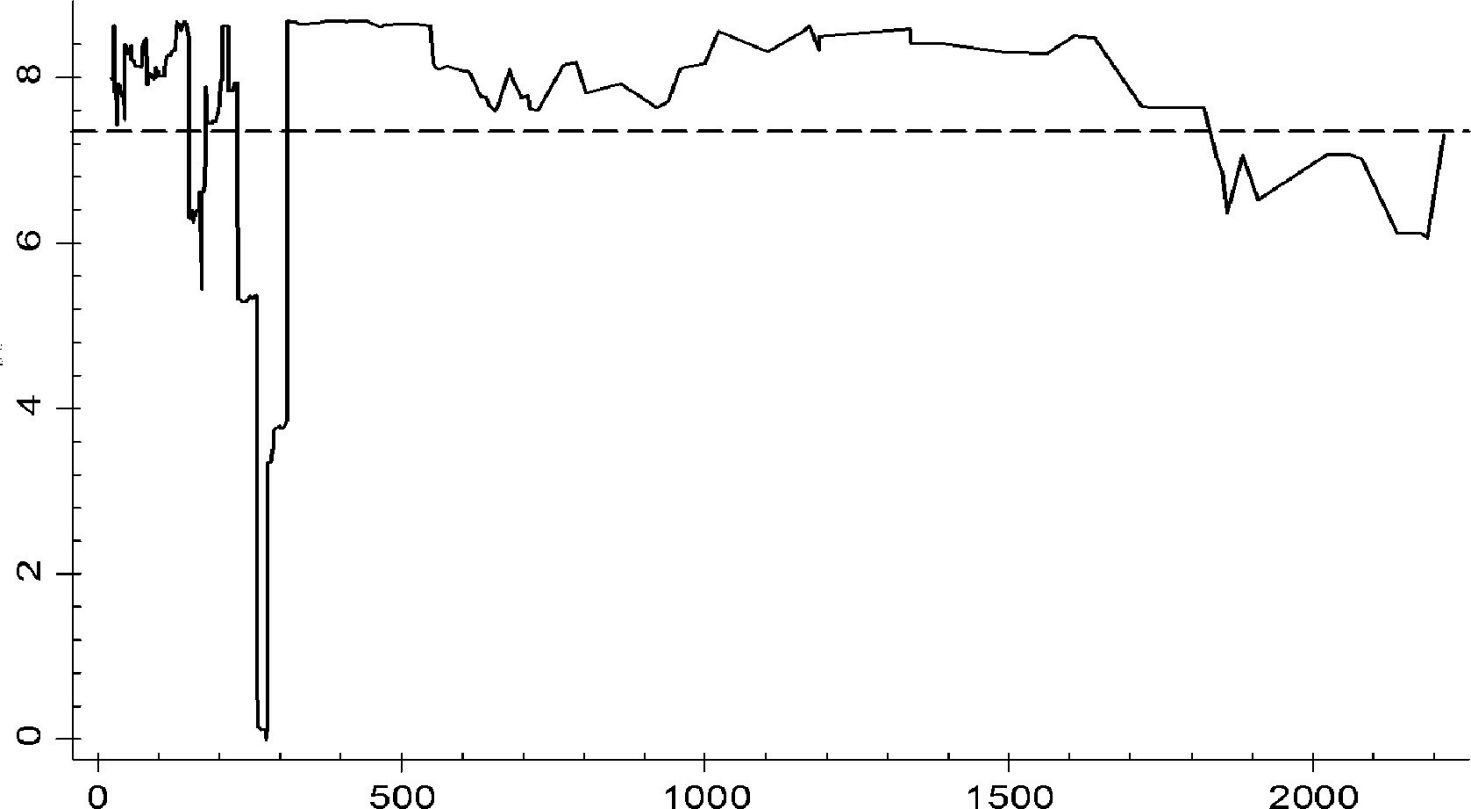
Visualization of threshold estimation test results.

Fig 2 shows the results of threshold estimation test. Table 8 shows the results of parameter estimation. Only the estimated results of core explanatory variables and threshold values are reported. Fig 2 shows that when R&D capital stock is taken as the threshold variable, the LR value of the threshold estimate is far less than the critical value 7.35 at the significance level of 5%. Therefore, the null hypothesis is accepted, indicating that the threshold estimate is true and effective. Table 8 shows the estimated threshold value is 276.816, meaning if R&D capital stock is less than 276.816, the impact of skill biased technical progress on the employment of skilled labor in manufacturing is not significant. Once the R&D capital stock exceeds the threshold estimated value of 276.816, the impact will become significant. We take Chow test to verify the significance level of the threshold. Ho: no Structural Change, the Chow Test is 3.97,P-Value > F(2, 234) = 0.017, which means the threshold is significant. To be specific, technical progress is biased towards skilled labor for 1 unit, the skilled labor employment will increase by about 4.5% at the significance level of 5%. Further, we calculated the mean value of R&D capital stock in various manufacturing industries during the sample period and classified the manufacturing industries base on the threshold estimate. The results shows there are 12 industries exceeded the threshold value 276.816. They are Textiles, chemical raw materials and chemical products manufacturing industry, pharmaceutical manufacturing industry, nonmetal mineral products, ferrous metal smelting and rolling processing industry, non-ferrous metal smelting and rolling processing industry, fabricated metal products, general equipment manufacturing industry, special equipment manufacturing, transportation equipment manufacturing, electric machinery and equipment manufacturing, communications equipment, computers and other electronic equipment manufacturing industry. The remaining 16 sectors were below the threshold, accounting for about 57 percent of the total. So, for most industries, the R&D capital accumulation level is still insufficient.

## 5. Conclusion and suggestions

This paper analyzes the biased technical progress and tests the impact of technical progress bias on employment empirically based on China’s manufacturing data. The empirical results show that, the technical progress of 16 industries is in favor of skilled labor, while the remaining 12 industries is in favor of unskilled labor. Technical progress has a positive impact on the total manufacturing employment and significant positive effect on the unskilled labor’s employment, but no significant impact on skilled labor’s employment in manufacturing. Further inspection found there is a threshold effect about the impact of skill biased technical progress on the employment of skilled labor: taking the industry value-added per capita (R&D capital stock) as threshold variable, if they did not pass the threshold, skill biased technical progress will have no significant impact on skilled labor’s employment; If the industry value-added per capita (R&D accumulation level) pass the threshold, the impact will be significant and positive.

However, the average value of the industry value-added per capita (R&D capital stock) in most industries of China’s manufacturing did not pass the threshold during the sample period, so the impact of skill biased technical progress on the skilled labor’s employment became insignificant. So, for the government departments, they should realize that technical progress and employment are not necessarily mutually exclusive. If appropriate technologies are selected, technical progress and employment growth can be coordinated. The empirical study of this paper found that the technical progress bias plays a positive role in promoting the total employment of China’s manufacturing. Therefore, the government should face the new technology with a more positive attitude, seize the historical opportunity of the new round of technological change and help manufacturing enterprises to carry out technological transformation in various ways.

## Supporting information

S1 Appendix Appendix including Tables 1A, 4A, 5A and 7A. (DOCX)
