# Expression of concern: Will advancement in technologies bring fear and damage human employment? Evidence from China’s manufacturing industry

**DOI:** 10.1371/journal.pone.0343791

**Published:** 2026-02-26

**Authors:** The PLOS One Editors

The *PLOS One* Editors issue this Expression of Concern to alert readers that this article [1] was identified as one of a series of submissions for which we have concerns about adherence to the journal’s authorship policy. We regret that these issues were not addressed prior to the article’s publication.
