# Supplementary material for: Will advancement in technologies bring fear and damage human employment? Evidence from China’s manufacturing industry
Source: PLoS One. 2024 Apr 26;19(4):e0295942. doi: 10.1371/journal.pone.0295942 (PMC11051635; doi:10.1371/journal.pone.0295942)
Supplement: S1 Appendix — (DOCX) [file pone.0295942.s001.docx]

**Appendix-Supporting Information**

Tables:

Table 1A: Descriptive statistics of relevant variables

| **Variable name** | **Sign** | **Mean** | **Standard Deviation** | **Minimum** | **Maximum** |
| --- | --- | --- | --- | --- | --- |
| Output | lnY | 7.2163 | 1.0532 | 4.3561 | 9.9456 |
| Capital stock | lnK | 7.5433 | 0.9698 | 4.9237 | 9.8195 |
| Skill labor | lnH | 3.8997 | 1.1432 | 0.8193 | 6.0773 |
| Unskilled labor | lnL | 7.3143 | 0.8386 | 5.1738 | 8.9643 |

Table 4A: The bias of technical progress of China's manufacturing

| **Bias toward**  **skilled labor** | Agricultural food processing industry, textile industry, furniture manufacturing, oil processing and coking and nuclear fuel processing industry, chemical raw materials and chemical products manufacturing industry, pharmaceutical manufacturing, rubber products, plastic products, non-metallic mineral products, ferrous metal smelting and rolling processing industry, non-ferrous metal smelting and rolling processing industry, fabricated metal products, general equipment manufacturing industry, special equipment manufacturing, transportation equipment manufacturing industry, communications equipment, computers and other electronic equipment manufacturing |
| --- | --- |
| **Bias toward**  **unskilled labor** | Food manufacturing industry, beverage manufacturing industry, tobacco products, textile and garment, shoes, hats manufacturing industry, leather, fur, and feathers (fine hair) and its products, wood processing and wood, bamboo, cane, palm, grass products, and paper and paper products, printing and duplicate record medium, culture and education sporting goods manufacturing industry, chemical fiber industry, electrical machinery and equipment manufacturing, instruments, office machinery manufacturing and culture |

Table 5A: Descriptive statistics of relevant variables

| **Variable name** | **Sign** | **Mean** | **Standard Deviation** | **Minimum** | **Maximum** |
| --- | --- | --- | --- | --- | --- |
| Total Employment | lnL | 7.3553 | 0.8413 | 5.2263 | 9.0113 |
| Employment of  Skilled Labor | lnS | 3.8997 | 1.1432 | 0.8193 | 6.0773 |
| Employment of  unskilled Labor | lnUS | 7.3143 | 0.8386 | 5.1738 | 8.9643 |
| Technical progress bias index | DBias | 0.0039 | 1.6022 | -6.7888 | 10.3743 |
| Wages for all | lnW | 2.6801 | 0.4655 | 1.6882 | 4.2636 |
| Wage of  Skilled labor | lnWS | 3.0182 | 0.5554 | 1.5245 | 4.3111 |
| Wage of  unskilled labor | lnWUS | 2.6636 | 0.4627 | 1.6886 | 4.2698 |
| Output | lnY | 7.2163 | 1.0532 | 4.3561 | 9.9456 |
| Capital Stock | lnK | 7.5433 | 0.9698 | 4.9237 | 9.8195 |
| Import | lnIM | 5.9666 | 1.7581 | 0.8177 | 9.5612 |
| Export | lnEX | 6.5055 | 1.5931 | 1.2092 | 9.8103 |
| R&d capital stock | lnRD | 5.3246 | 1.2681 | 2.5312 | 8.0552 |
| Foreign direct investment | lnFDI | 6.2334 | 1.0999 | 2.5930 | 9.0198 |

Table 7A: Threshold effect test results

| **Model** | **F Value** | **P Value** | **Sample** | **1%** | **5%** | **10%** |
| --- | --- | --- | --- | --- | --- | --- |
| Single threshold | 5.877** | 0.027 | 300 | 7.974 | 5.004 | 3.774 |
| Double threshold | 3.789 | 0.120 | 300 | 10.694 | 5.704 | 4.445 |
| Triple threshold | 3.979 | 0.117 | 300 | 9.954 | 7.137 | 4.518 |

Notes: Levels of significance: ***1%, **5%, *10%.The P values and critical values were obtained by repeated sampling for 300 times
